# Extreme climatic events to intensify over the Lake Victoria Basin under global warming

**DOI:** 10.1038/s41598-023-36756-3

**Published:** 2023-06-15

**Authors:** Obed M. Ogega, Enrico Scoccimarro, Herbert Misiani, James Mbugua

**Affiliations:** 1 The African Academy of Sciences, Nairobi, Kenya; 2 Department of Chemistry and Physics, Mount Saint Vincent University, Halifax, NS Canada; 3 Fondazione Centro Euro-Mediterraneo Sui Cambiamenti Climatici, 40127 Bologna, Italy; 4 IGAD Climate Prediction and Applications Centre, Nairobi, Kenya; 5 Jomo Kenyatta University of Agriculture and Technology, Nairobi, Kenya

**Keywords:** Atmospheric science, Climate change

## Abstract

This paper presents an analysis of future precipitation patterns over the Lake Victoria Basin, East Africa, using bias-corrected CMIP6 model projections. A mean increase of about 5% in mean annual (ANN) and seasonal [March–May (MAM), June–August (JJA), and October–December (OND)] precipitation climatology is expected over the domain by mid-century (2040–2069). The changes intensify towards the end of the century (2070–2099) with an increase in mean precipitation of about 16% (ANN), 10% (MAM), and 18% (OND) expected, relative to the 1985–2014 baseline period. Additionally, the mean daily precipitation intensity (SDII), the maximum 5-day precipitation values (RX5Day), and the heavy precipitation events—represented by the width of the right tail distribution of precipitation (99p–90p)—show an increase of 16%, 29%, and 47%, respectively, by the end of the century. The projected changes have a substantial implication for the region—which is already experiencing conflicts over water and water-related resources.

## Introduction

During late 2019 and early 2020, unprecedented high lake water-levels were observed in Lake Victoria, resulting in massive flooding in the lake-adjacent areas^1^. The high-water-levels re-ignited the scientific debate on the impacts of a changing climate on Lake Victoria’s water budget. Indeed, the impacts of a changing climate in East Africa^2,3^ on the biodiversity of the Lake Victoria Basin (LVB) are already being observed at multiple biological levels ranging from genes to biomes. Some of these impacts include enhanced spatio-temporal precipitation variability, a reduction in biodiversity (including fish), periodical lake water-level and quality fluctuations, and dwindling crop yields and emergence of crop diseases in the lake-adjacent farming areas^4,5^. Given that the lake’s fishes, and biodiversity in general, are significantly sensitive and vulnerable to climate change, adequate monitoring and appropriate conservation actions are required to minimize the impacts of climate change on the lake’s biodiversity.

Some work has been done towards understanding precipitation patterns and associated water-level fluctuations over Lake Victoria and its basin. For instance, the lake has been found to be notorious with intense lightning and convective storms^6^. The lake’s severe weather and water currents have also been linked to boat accidents linked to approximately 5000 deaths on the lake annually^7,8^. However, the understanding of weather and climate dynamics in the LVB remains limited due to, in part, the inability of current climate models to adequately resolve weather and climate dynamics over Lake Victoria^9–11^. This paper complements earlier works by using bias-corrected model data from the sixth phase of the Coupled Model Intercomparison Project (CMIP6^12^) to generate the most realistic estimates of future precipitation patterns over the study domain.

## Methods

### Study area

**Figure 1:**
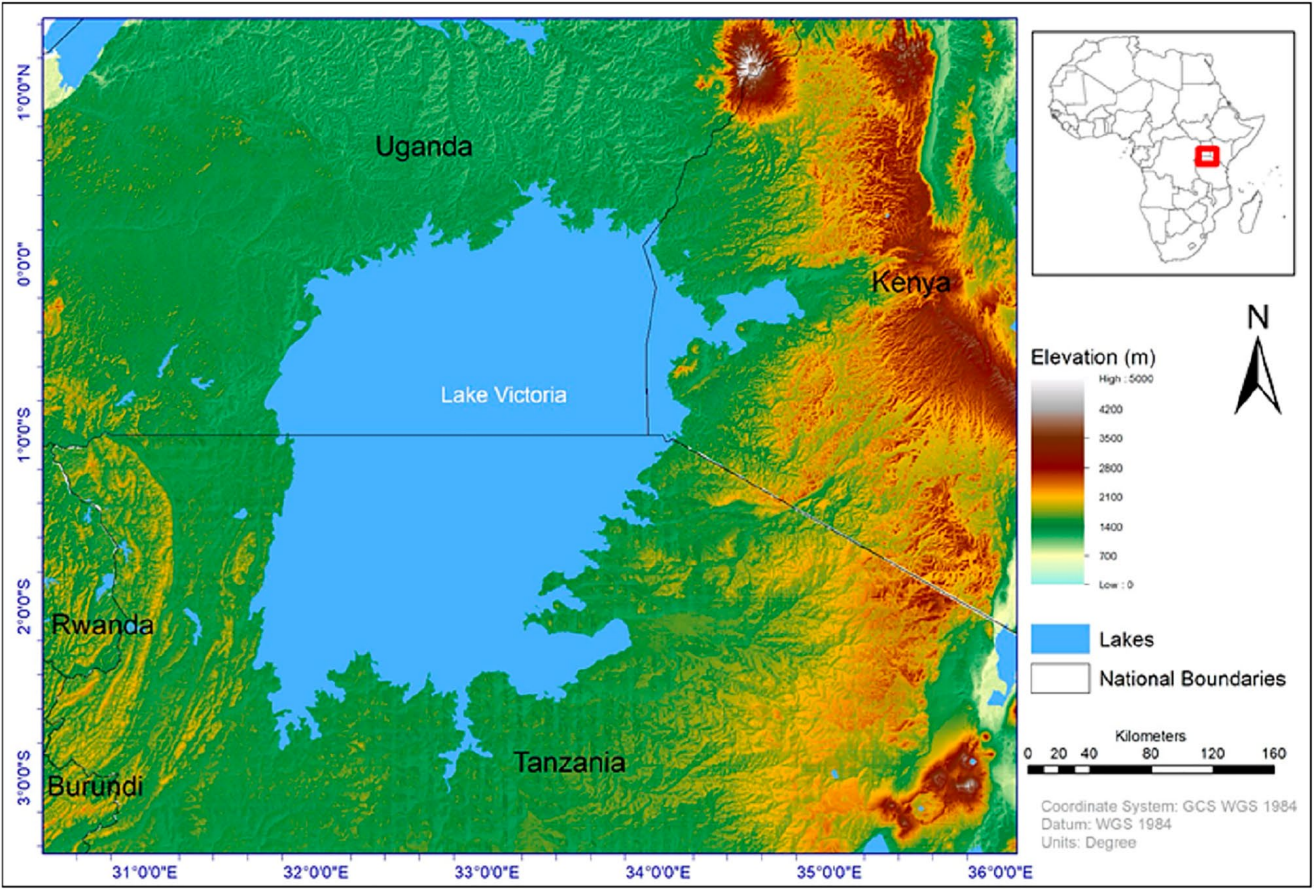
A topographic map of the study domain produced using three primary layers, namely, elevation data sourced as a 30 × 30-m raster file from the United States Geological Survey (USGS) archive (https://earthexplorer.usgs.gov/), administrative boundaries sourced from the Global Administrative Areas (GADM) archive (www.gadm.org/download), and water bodies sourced from the World Bank archives (https://datacatalog.worldbank.org/search/dataset/0040797). All cartographical work was performed in ArcGIS Desktop 10.8.1 version 10.8.14362 (https://www.esri.com/).

The study focussed on Lake Victoria and its immediate adjacent catchment area (Fig. 1). The lake is located at an altitude of about 1135 m above sea-level, spanning an area of about 68,000 square kilometres^13^. It is Africa’s largest freshwater body and the world’s largest inland fishery supporting at least 40 million inhabitants^14^. The lake and its resources fuel East Africa’s economy with the lake’s catchment providing about 90% of hydropower for Uganda, Burundi, and Rwanda. The lake’s basin (LVB) is renowned for its richness and endemism in species^4,15,16^. It has massive natural resources including forests, fisheries, rangelands, and wetlands from which communities in and around the basin draw their livelihoods. During the dry season over the Ethiopian highlands from whence the Nile’s main tributary (the Blue Nile) comes, the White Nile—the only outlet for Lake Victoria—contributes about 70% of all water reaching Egypt^17^.

The LVB region experiences complex meteorological and climatic conditions influenced by local and large-scale systems. For instance, the region is punctuated with large and small lakes that strongly modify the mesoscale atmospheric dynamics by creating a thermal contrast with the adjacent land areas. The thermal contrast contributes to convection over the lake during the night while restraining it during the day^18,19^. Additionally, the katabatic and anabatic winds induced by the mountainous areas in the LVB modify precipitation characteristics^20^ while storms on land influence the region’s temperature, humidity, and atmospheric conditions—resulting in strong nightly storms over Lake Victoria^19^. The mountainous areas in and around the LVB strongly influence the flow of large-scale moisture-carrying winds, resulting in modulated precipitation patterns^9,21^. Modelling these complex local and large-scale systems, whose understanding is still limited^22^, is quite challenging and efforts are underway to improve their representation in regional and global models^22^.

### Data

Three gridded data products were used as reference datasets in the current study, including the Climate Hazards Group InfraRed Precipitation with Station data, version 2.0 (hereinafter CHIRPS). CHIRPS is a quasi-global precipitation dataset available from 1981 to near present, spanning 50°S–50°N and all longitudes. It incorporates climatology, in-situ station data, and satellite imagery to form a gridded precipitation time series^23^. The current study used the monthly 0.25° by 0.25° resolution dataset, downloaded from https://www.chc.ucsb.edu/data.

The second dataset used is the Multi-Source Weighted-Ensemble Precipitation version 2.8 (hereinafter MSWEP), downloaded from https://www.gloh2o.org/mswep/. MSWEP is a global 3-hour 0.1° resolution precipitation product that merges gauge, reanalysis, and satellite data to obtain high quality precipitation estimates across the globe^24^. Lastly, we used the Global Precipitation Climatology Centre (GPCC) full data monthly product version 2020 (v2020) at 0.5° by 0.5° resolution, obtained from the NOAA PSL, Boulder, Colorado, USA via https://psl.noaa.gov. The full data product is based on quality-controlled data from 67,200 observation stations worldwide. The dataset contains monthly totals on a regular grid with precipitation anomalies at the stations interpolated before being superimposed on the GPCC climatology v2020 in the corresponding resolution^25^. These datasets have been validated and used widely over the study domain (e.g.,^2,26,27^).

To assess future precipitation patterns over the study domain, we used 17 bias-corrected general circulation model (GCM) datasets (Table 1) from the NASA Earth Exchange Global Daily Downscaled Projections (NEX-GDDP-CMIP6). The NEX-GDDP-CMIP6 datasets are produced using a daily variant of monthly bias correction/spatial disaggregation method, resulting in a 0.25-degree horizontal product^28^. They are downscaled historical and future (spanning the period 1950–2100) climate projections based on CMIP6 model output, including datasets from the ScenarioMIP model runs^29,30^ that are based on the Shared Socioeconomic Pathways (SSPs^31^) Simulations from the ScenarioMIP provide the basis for investigating various science and policy questions particularly relevant to scenario-based analysis such as the role of specific forcings^29^. The current study used projections under the SSP5-8.5 emission scenario due to its ability (unlike lower emission scenarios) to reproduce global warming projections exceeding 2.0 °C^32^.

**Table 1:** NEX-GDDP-CMIP6 datasets used in the study. All the data have a spatial resolution of 0.25 × 0.25 degrees and are based on SSP5-8.5.

Model ID (Variant) [Abbreviation]	Institution	Main references
CMCC-ESM2 (r1i1p1f1) [CMCC]	Centro Euro-Mediterraneo sui Cambiamenti Climatici (CMCC), Italy	^33^
CNRM-ESM2-1 (r1i1p1f2) [CNRM]	Centre National de Recherches Météorologiques (CNRM) and Centre Européen de Recherche et de Formation Avancée en Calcul Scientifique (CERFACS), France	^34^
EC-Earth3 (r1i1p1f1) [EC]	Consortium Europe	^35^
FGOALS-g3 (r3i1p1f1) [FGOALS]	Chinese Academy of Sciences (CAS), China	^36^
GFDL-ESM4 (r1i1p1f1) [GFDL]	National Oceanic and Atmospheric Administration, Geophysical Fluid Dynamics Laboratory (NOAA), USA	^37^
IPSL-CM6A-LR (r1i1p1f1) [IPSL]	Institut PierreSimon Laplace (IPSL), France	^38^
KACE-1-0-G (r1i1p1f1) [KACE]	National Institute of Meteorological Sciences, Korea Meteorological Administration (NIMS-KMA), Republic of Korea	^39^
MIROC-ES2L (r1i1p1f2) [MIROC]	Japan Agency for Marine-Earth Science and Technology, Atmosphere and Ocean Research Institute, Japan	^40^
MIROC6 (r1i1p1f1) [MIROC6]		^41^
MPI-ESM1-2-HR (r1i1p1f1) [MPI_HR]	Max Planck Institute for Meteorology (MPI), Germany	^42^
MPI-ESM1-2-LR (r1i1p1f1) [MPI_LR]		^43^
MRI-ESM2-0 (r1i1p1f1) [MRI]	Meteorological Research Institute (MRI), Japan	^44^
NESM3 (r1i1p1f1) [NESM3]	Nanjing University of Information Science and Technology (NUIST), China	^45^
NorESM2-LM (r1i1p1f1) [NorESM2_LM]	NorESM Climate Modelling Consortium (NCC), Norway	^46^
NorESM2-MM (r1i1p1f1) [NorESM2_MM]		
TaiESM1 (r1i1p1f1) [TaiESM1]	Research Center for Environmental Changes, Academia Sinica Taiwan (AS-RCEC), China	^47^
UKESM1-0-LL (r1i1p1f2) [UKESM1]	Met Office-NERC, United Kingdom	^48^

More information about the models is available at https://esgf-node.llnl.gov/search/cmip6/.

### Analysis

First, we assessed the performance of the bias-corrected CMIP6 model simulations in reproducing precipitation patterns over the study domain, relative to observations. Here, we computed differences in mean spatial and temporal values between observations and model simulations. The performance assessment was done with reference to the 1985–2014 baseline corresponding to the last 30 years of the CMIP6 historical runs which cover the period 1850–2014. The 1985–2014 period is widely used in CMIP6 projection analysis studies (e.g.,^49,50^).

Secondly, an assessment of future precipitation patterns over the study domain was done by calculating differences in mean values between the future period and the control period (1971–1999), using the annual precipitation indices and statistics presented in Table 2. Here, the periods 2020–2049 and 2070–2099 were used to represent the “near future (MID)” and “future (FUT)”, respectively.

**Table 2:** A list of annual precipitation indices and annual precipitation statistics used in the current study.

Descriptor	Acronym	Description
Simple precipitation intensity index	SDII	Mean precipitation amount on a wet day. Let RR_*ij*_ be the daily precipitation amount on wet day w (RR ≥ 1 mm) in period *j*. If *W* represents the number of wet days in *j* then the simple precipitation intensity index SDII_j_ = sum (RR_*wj*_)/W
Max 5‐day precipitation amount	Rx5day	Maximum consecutive 5‐day precipitation
Width of the right tail distribution of precipitation	99p–90p	Calculated as the difference between the 99th and the 90th percentiles (99p–90p), where 90p defined as follows; for every adjacent sequence *t*_1,…, *t_n* of timesteps of the same year, 90p is given by *o(t, x)* = *p*th percentile{*i(t’, x), t*_*1*_ < *t’* ≤ *t*_*n*_}; here computed for the 90th percentile. For this study, 90p represents the threshold for identifying heavy precipitation events 99p represents very intense precipitation events and is defined as; for every adjacent sequence *t*_1, …, *t_n* of timesteps of the same year, 99p is given by *o(t, x)* = *p*th percentile{*i(t’, x), t*_*1*_ < *t’* ≤ *t*_*n*_}; here computed for the 99th percentile

Additionally, we computed mean annual (ANN) and seasonal (MAM, JJA, and OND) precipitation values over the study domain. We also used the 99p–90p statistic (Table 2) to represent the right tail of precipitation distribution over the study domain^51^. Here, 90p and 99p were calculated by accumulating daily precipitation values over each grid point with 99p and 90p, representing very intense and heavy precipitation events, respectively. The percentiles for 99p and 90p were calculated for all days in the data.

All statistical computations were done on the native data grids before bilinearly interpolating the observational datasets (with relatively finer resolutions than CMIP6 model data) onto CMIP6 model data grids to facilitate comparison as described in^52,53^. When computing differences in mean values for various variables, we used the Student’s *t* test^54^ to show significant values at 99% confidence level.

## Results and discussion

### Validation of CMIP6 historical simulations over LVB

**Figure 2:**
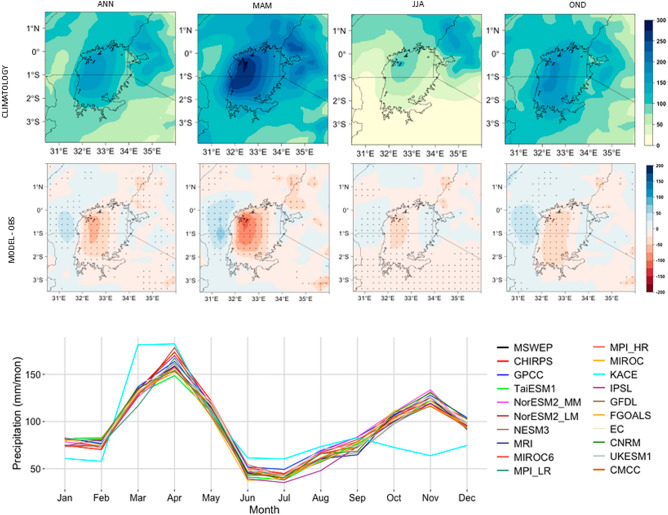
Precipitation climatology over the study domain using MSWEP data (top panel), differences in mean precipitation between an ensemble mean of the top five performing models (mid panel), and annual cycles for all the three reference datasets and all the 17 model datasets (bottom panel). Stippling (mid panel plots) shows significant values at 99% confidence interval. All units are in mm/month.

A plot of precipitation climatology over the study domain (Fig. 2, top panel) shows MAM to be the wettest season followed by OND. Relatively more precipitation is recorded on Lake Victoria than the onshore areas, with the western part of the lake showing a wetter regime than the rest of the lake during MAM and OND seasons. A similar pattern is recorded for the OND season, except with lower precipitation values than those of the MAM season. The JJA season is relatively dry with most of the domain recording less than 50 mm/month of precipitation. During this season, the southern part of the domain is drier than the northern part, with precipitation values over parts of western Kenya (north-east of the study domain) exceeding 150 mm/month. Annually (ANN), most of the study domain records at least 100 mm/month with areas over the lake and western Kenya recording the highest values. The highest mean precipitation values (up to 300 mm/month) are recorded in the MAM season while the least values (less than 50 mm/month) are recorded in JJA.

A plot of the precipitation climatology over the study domain showed no discernible differences between the three reference datasets (Supplementary Information, Fig. S 1). MSWEP and CHIRPS data showed similar results, with GPCC showing marginally wetter conditions. Hence, MSWEP was chosen as the reference dataset for the model validation part of the study. Here, models generally showed a dry bias relative to MSWEP data with biases ranging roughly between -50 and 50 mm/month for most of the models (Fig. 2, mid panel; Supplementary Information, Figs. S 1, S 2, S 3, S 4 and S 5). The greatest biases (below-100 mm/month) were recorded over the western part of Lake Victoria (around 0.5° South and 32.3° East). However, the biases recorded for most models were insignificant (at 99% confidence level) over most of the domain. Further, an ensemble mean for the top-five models with the least biases showed a better performance than an ensemble mean of all the 17 models used in the study. The top five models (CMCC, CNRM, GFDL, MPI_LR, and NorESM2_LM) were selected for featuring among the top performers per category (Supplementary Information, Figs. S 1, S 2, S 3, S 4 and S 5).

Apart from KACE, all other models reproduced the study domain’s annual cycles in concert with the reference datasets (Fig. 2, bottom panel). Notably, our results show a departure from the traditional rainy seasons; extending the MAM season to include February and May (with a peak in April), and the OND season to begin in September with a peak in November. This observation points to a potential influence of Lake Victoria, which tends to have a different climatology than its neighbouring areas^55,56,^, on the study domain's climatology.

Generally, the bias-corrected CMIP6 model data used in the current study could reproduce the study domain’s climatology with good skill. The observed minimal biases were expected due to the relatively coarse spatial resolution of CMIP6 model outputs as well as the study area’s strong local climate systems and mesoscale convective systems^9,57^. Additionally, the skill of the current climate models is limited by the current understanding of the climate system (including the interactions between the atmosphere and the ocean) coupled with limitations in computational technology^10^.

### Analysis of future precipitation patterns over Lake Victoria Basin

**Figure 3:**
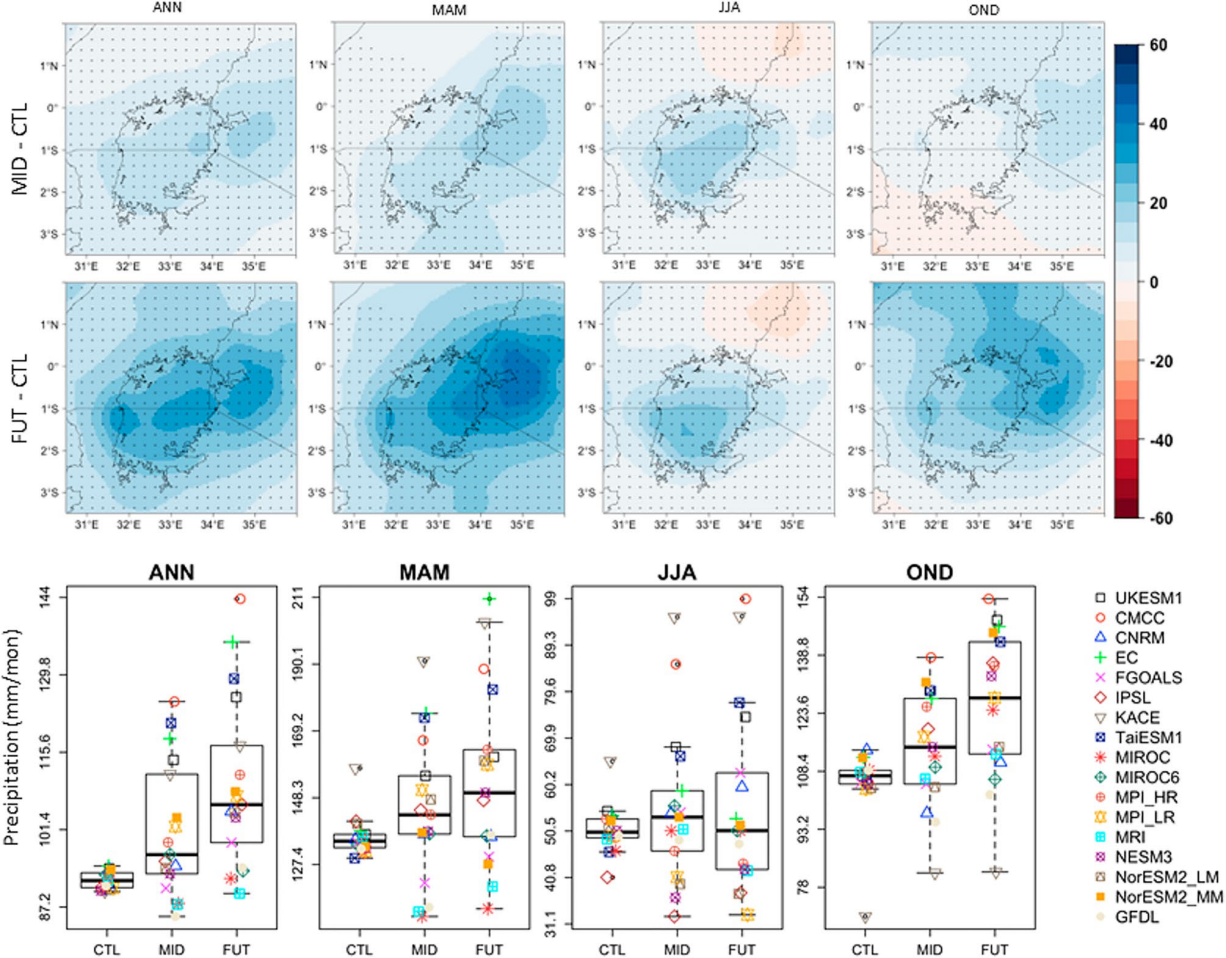
Changes in mean annual (ANN) and seasonal (MAM, JJA, and OND) precipitation climatology for the period 2040–2069 (MID; top panel) and 2070–2099 (FUT; mid panel) relative to the 1985–2014 period (CTL), using an ensemble mean of the top-five models. Stippling (for spatial plots) shows significant values (at 99% confidence level). Bottom panel presents mean values for ANN, MAM, JJA, and OND averaged over the study domain for the CTL, MID, and FUT periods, for all the models. All units are in mm/month.

An analysis of future precipitation patterns over the study domain shows a general increase in mean precipitation for all the seasons considered (Fig. 3, top and mid panels). Most of the domain records significant changes in precipitation with more changes likely to occur over Lake Victoria and its surroundings than the rest of the study domain. More changes are shown for the end of the century period compared to the mid-century period, for all the seasons. All the models agree on the change signal for ANN (Supplementary Information, Fig. S 6) while 88% of the models agree on the signal (wet) for OND (Supplementary Information, Fig. S 9). A lower model agreement (70%) is seen for the MAM season compared to ANN and OND while JJA records a 52% model agreement (Supplementary Information, Fig. S 8).

A look at the precipitation patterns averaged over the study domain (Fig. 3, bottom panel) showed a general increase in precipitation for ANN, MAM, and OND. In the 2050s (MID), precipitation over the study domain would increase by about 5% for ANN, MAM, JJA, and OND, relative to the baseline period (CTL). More changes would be expected by the end of the century for ANN (16%), MAM (10%), and OND (18%), respectively. No discernible changes are recorded for JJA.

**Figure 4:**
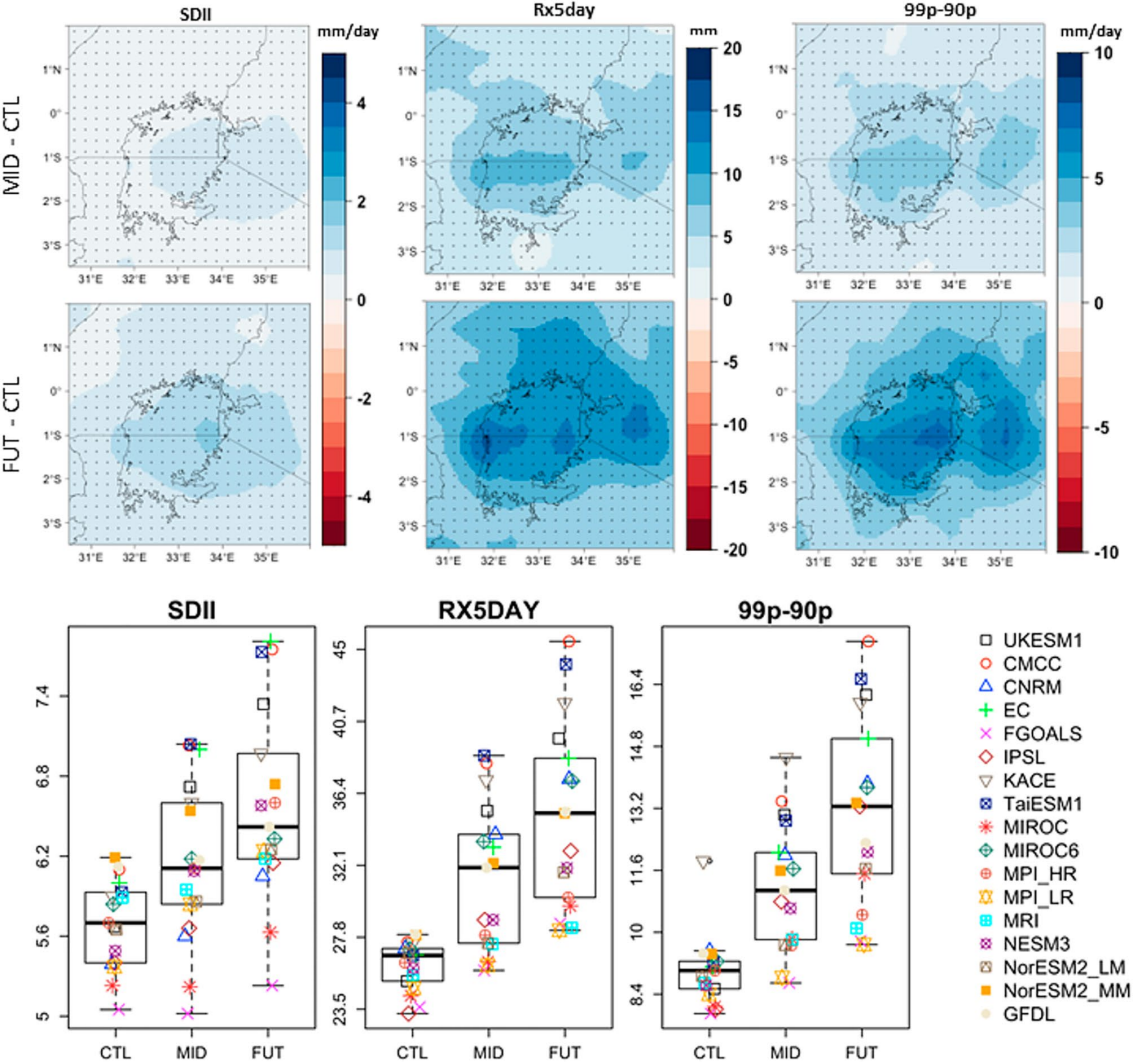
As in Fig. 3 but for SDII, Rx5day, and 99p–90p. SDII and 99p–90p are in mm/day while Rx5day is in mm.

At the daily timescale, all the three statistics analysed in our study (SDII, Rx5day, and 99p–90p) showed a significant increase by the mid and end-of-the-century periods (Fig. 4, bottom panel). Here, precipitation intensity (SDII) showed a potential increase of 6% and 16% by mid and end of the century, respectively. The changes were more pronounced over the eastern part of Lake Victoria and western Kenya (around 1° South and 34° East). Greater changes (18% and 29% for mid and end century periods, respectively) were recorded for the maximum 5-day precipitation (Rx5day). Just like in the SDII case, changes in Rx5day were more pronounced over Lake Victoria and its environs (centre of study domain). The 99p–90p statistic showed the greatest changes with a mean increase of about 22% and 47% for mid and end century periods, respectively. Again, the changes were more pronounced over Lake Victoria and its environs than the rest of the study domain.

The projected changes in precipitation patterns over the study area may be attributed to an increase in moisture content^9^ resulting from changes in local and mesoscale feedbacks^58^ as well as changes in mesoscale dynamics that affect thermal inertia of the lakes in the study area^9^. Additionally, increased warming may also alter large-scale atmospheric dynamics (such as patterns in moisture carrying lower tropospheric easterlies over the study area) and influence synoptic systems; potentially resulting in enhanced moisture convergence over the study domain^59,60^. We recommend a detailed detection and attribution study to enhance the understanding of potential drivers and their exact influence on the projected precipitation patterns over the LVB.

## Conclusion

Our findings indicate that more significant precipitation changes over the LVB are likely to occur at the sub-seasonal level compared to the seasonal and annual scales. Considering the relatively high sensitivity of our study domain to weather and climate change and variability, our findings contribute to the scientific evidence that can inform disaster risk reduction and climate change adaptation strategies for the study domain. Specifically, the projected increase in the width of the right tail precipitation distribution (99p–90p) and intensity (SDII) call for more robust measures to minimize disaster impact in a region where approximately 5000 people (mostly fishermen) perish annually on Lake Victoria from extreme weather-related accidents^8^. The projected increase in precipitation intensity, coupled with an intensification of hazardous thunderstorms over Lake Victoria^61,62^, call for adaptive capacity strengthening for fishermen and lake-adjacent communities most of whom are vulnerable to disaster risk associated with extreme weather and climate events^63^.

Moreover, the projected changes over Lake Victoria and its adjacent land areas were more pronounced than over other parts of the study area. In a region characterized by perennial weather-related disasters and extreme weather events, the projected changes may cause further disruption to the wellbeing of the region’s communities and systems. Additionally, the projected changes have a substantial implication for the study domain, and the larger Nile Basin Region, which is currently experiencing a rapid population density increase^64^ coupled with conflicts over water resources^65,66^. Hence, enhanced production and use of climate services is recommended to minimize the risk posed by the projected changes and, ultimately, enhance the socio-economic wellbeing of communities in the LVB. Results from the current study provide a basis for further research to enhance the understanding of climate change and variability over the LVB and inform adaptive capacity strengthening measures for the region.

## Supplementary Information

Supplementary Information.

## Data Availability

The bias-corrected CMIP6 datasets are available at https://www.nccs.nasa.gov/services/data-collections/land-based-products/nex-gddp-cmip6 while CHIRPS datasets can be downloaded from https://www.chc.ucsb.edu/data. MSWEP data is available at http://www.gloh2o.org/mswep/ and GPCC is available at https://psl.noaa.gov. Any other files are available on request from the corresponding author (O.M.O.).
