# Supplementary material for: Extreme climatic events to intensify over the Lake Victoria Basin under global warming
Source: Sci Rep. 2023 Jun 15;13:9729. doi: 10.1038/s41598-023-36756-3 (PMC10272193; doi:10.1038/s41598-023-36756-3)
Supplement: Supplementary file 1 — Supplementary Information. [file 41598_2023_36756_MOESM1_ESM.pdf]

Extreme climatic events to intensify in the Lake Victoria Basin under global warming

Supplementary information

Obed M. Ogega[a,b,*], Enrico Scoccimarro[c], Herbert Misiani[d], and James Mbugua[e]

List of figures

Fig. S 1: Climatology of the study domain for the period 1985-2014 1
Fig. S 2: Annual (ANN) climatology differences between model data and MSWEP data, for the period 1985-2014. Stippling shows significant values at 99% confidence level. All units are in mm/month 2
Fig. S 3: As in Fig. S 2 but for MAM 3
Fig. S 4: As in Fig. S 2 but for JJA 4
Fig. S 5: As in Fig. S 2 but for OND 5
Fig. S 6: Changes in mean annual (ANN) precipitation climatology for the period 2040-2069 (MID-CTLl) and 2070-2099 (FUT-CTL) relative to the 1985-2014 period (CTL), for all models and an ensemble mean for the top-five models identified in subsection 3.1 (ENStop5). Stippling shows significant values (at 99% confidence level). All units are in mm/month 6
Fig. S 7: As in Fig. S 6 but for MAM 7
Fig. S 8: As in Fig. S 6 but for JJA 8
Fig. S 9: As in Fig. S 6 but for OND 9
Fig. S 10: Changes in the maximum 5-day precipitation (Rx5day) climatology for the period 2040-2069 (MID-CTLl) and 2070-2099 (FUT-CTL) relative to the 1985-2014 period (CTL), for all models and an ensemble mean for the top-five models (ENStop5). Stippling shows significant values at 99% confidence level. All units are in mm ... 10
Fig. S 11: As in Fig. S 10 but for SDII 11
Fig. S 12: As in Fig. S 10 but for 99p90p. Units are in mm 12

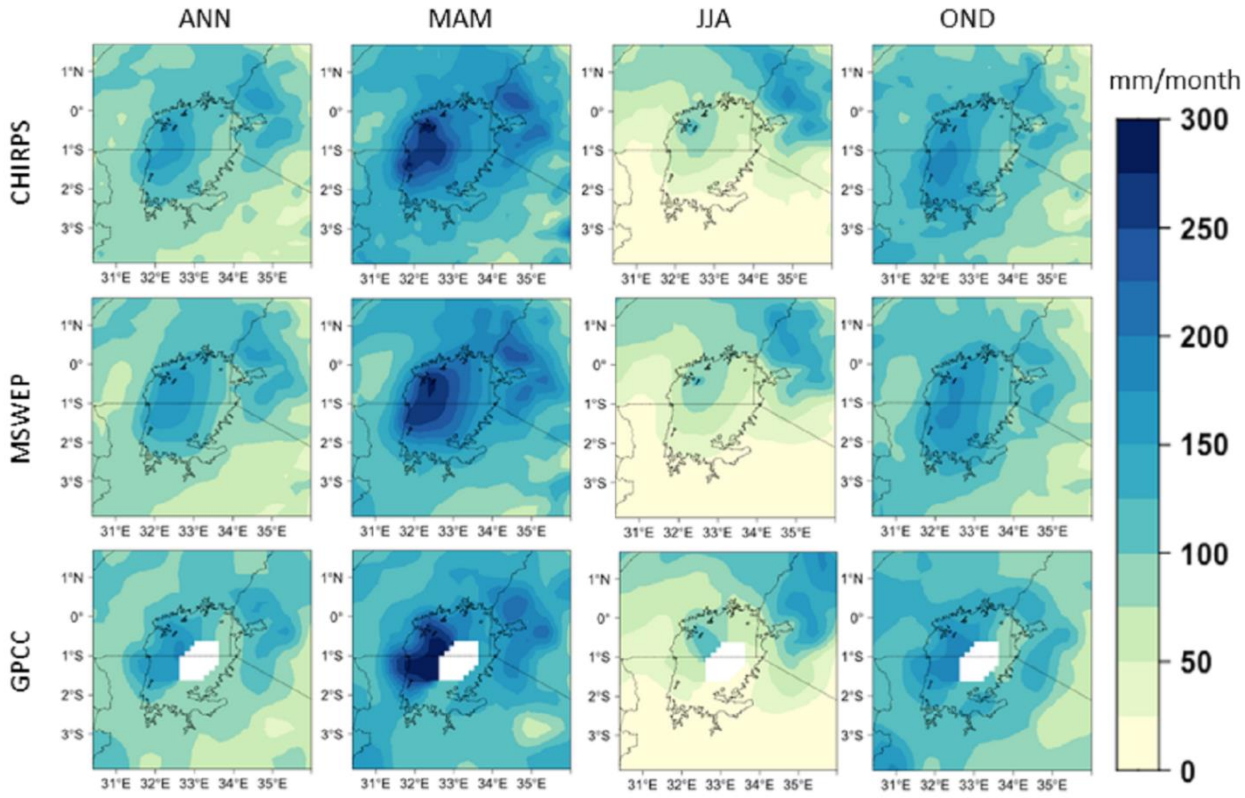

Fig. S 1: Climatology of the study domain for the period 1985-2014

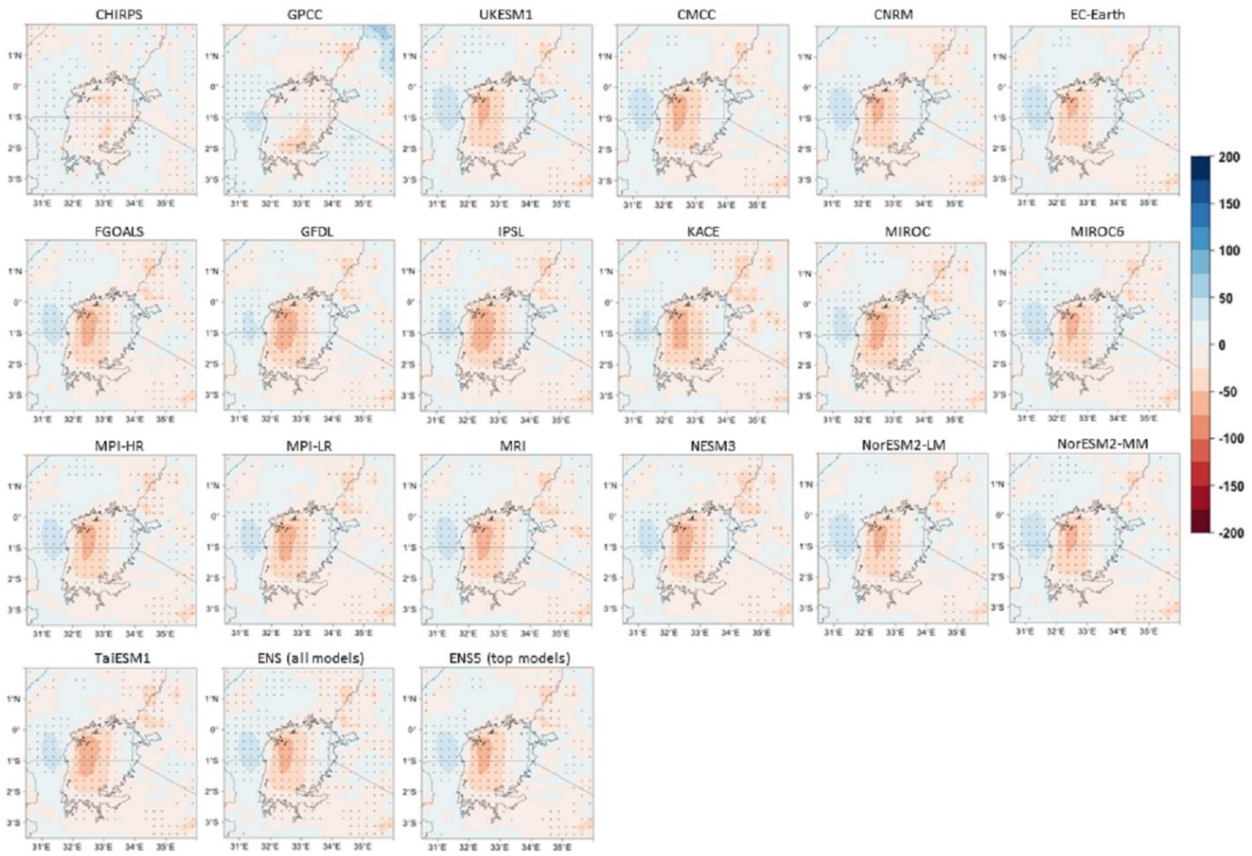

Fig. S 2: Annual (ANN) climatology differences between model data and MSWEP data, for the period 1985-2014. Stippling shows significant values at 99% confidence level. All units are in mm/month

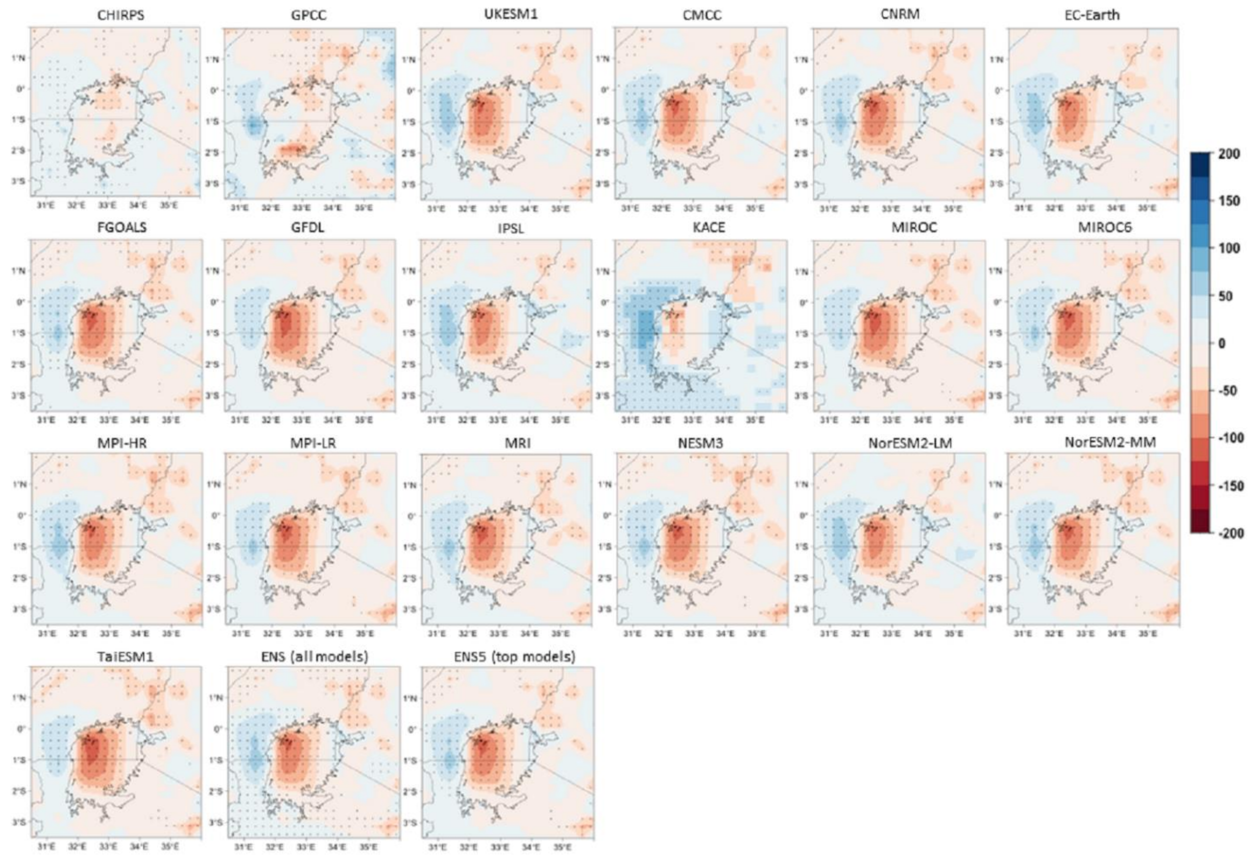

Fig. S 3: As in Fig. S 2 but for MAM

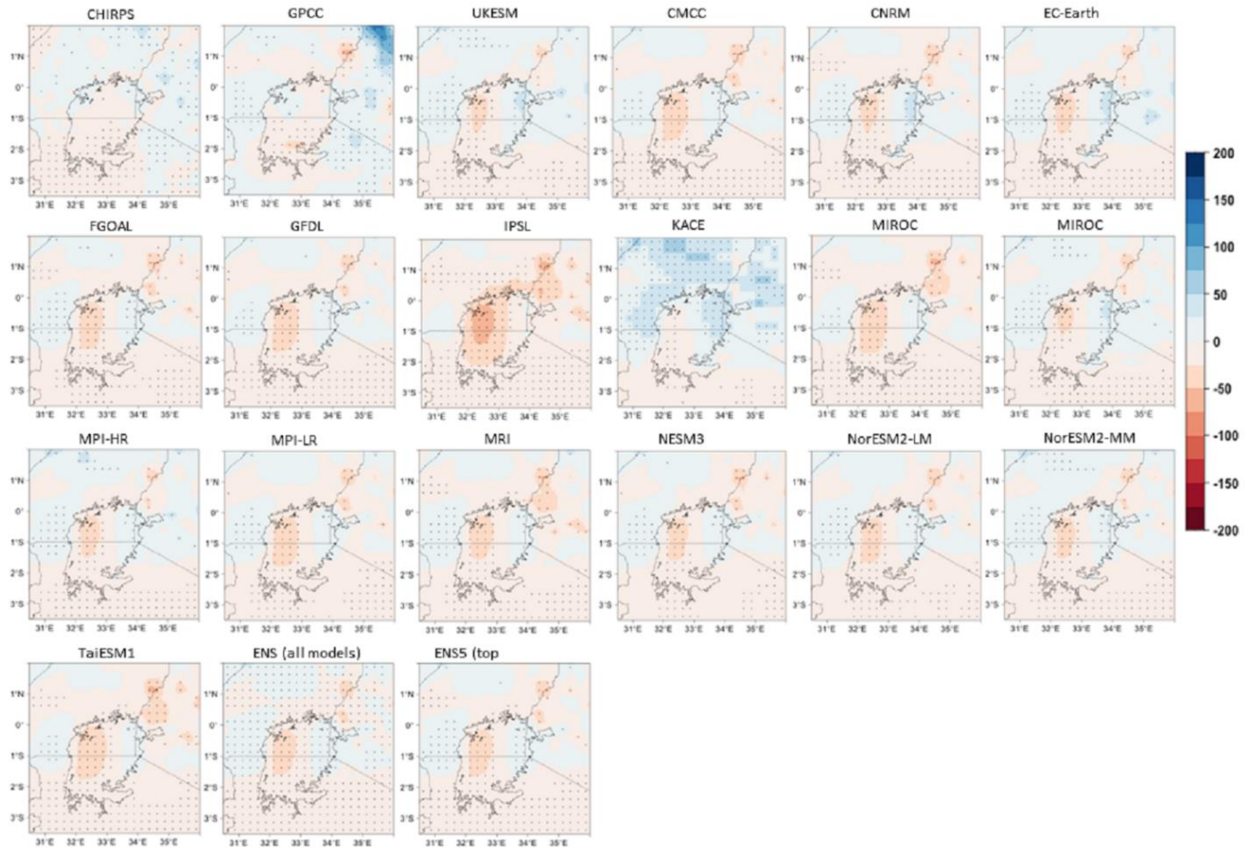

Fig. S 4: As in Fig. S 2 but for JJA

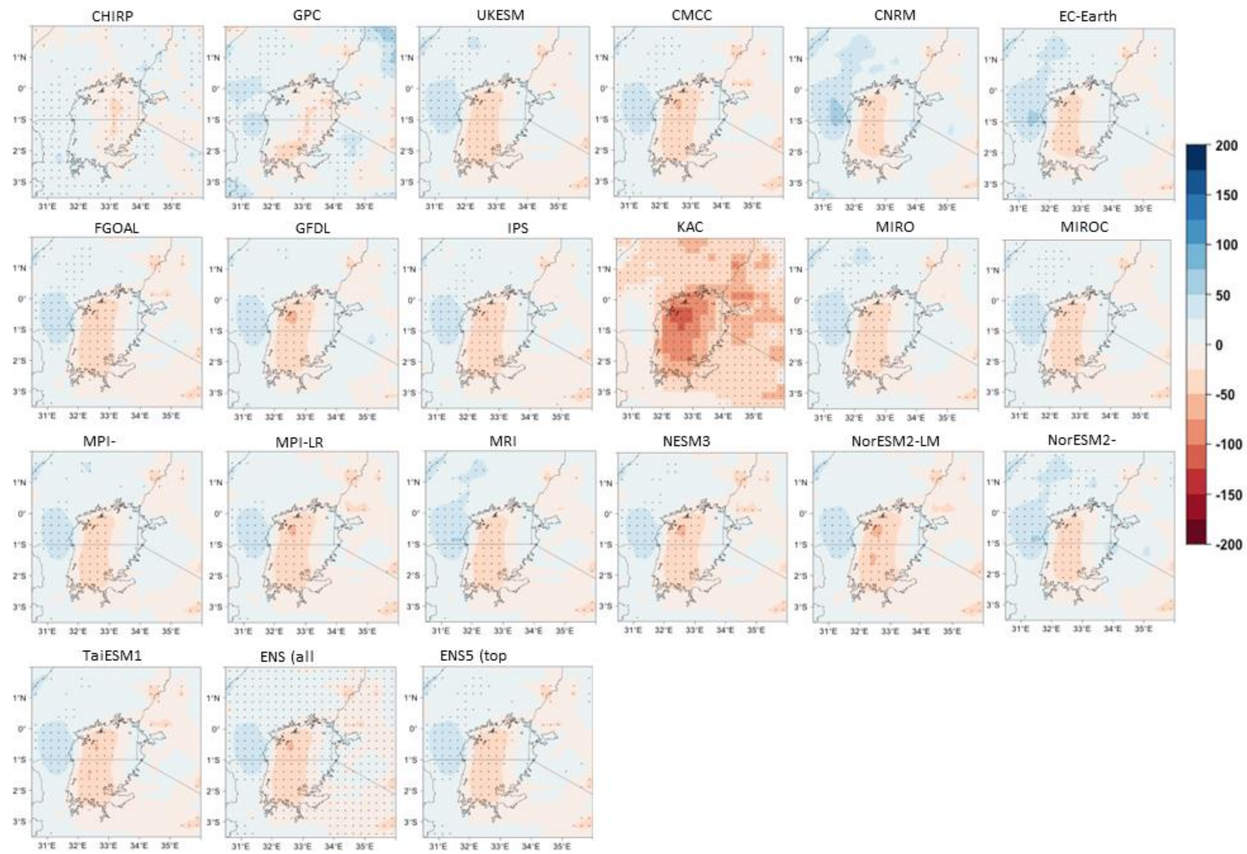

Fig. S 5: As in Fig. S 2 but for OND

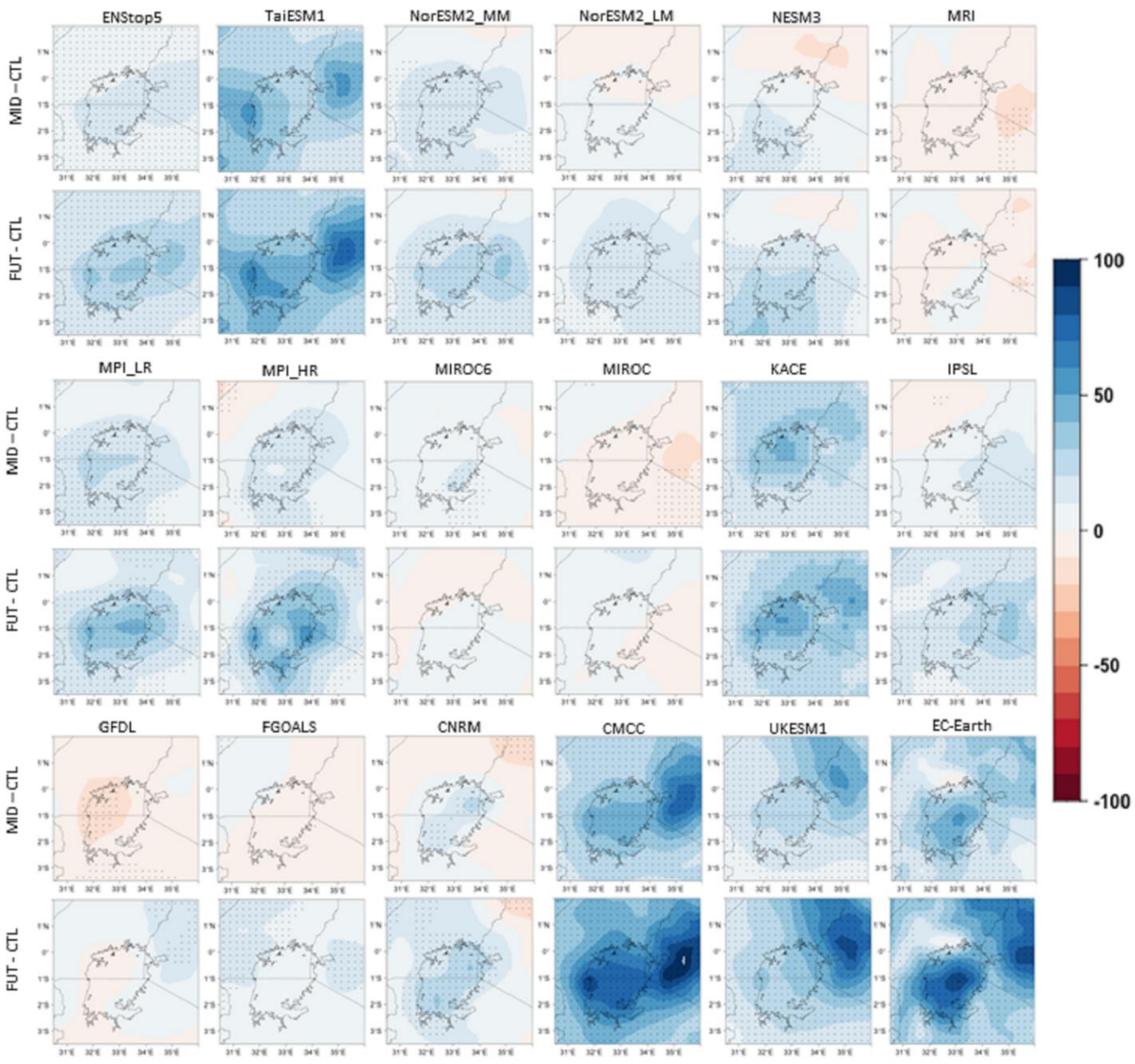

Fig. S 6: Changes in mean annual (ANN) precipitation climatology for the period 2040-2069 (MID-CTLl) and 2070-2099 (FUT-CTL) relative to the 1985-2014 period (CTL), for all models and an ensemble mean for the top-five models identified in subsection 3.1 (ENStop5). Stippling shows significant values (at 99% confidence level). All units are in mm/month

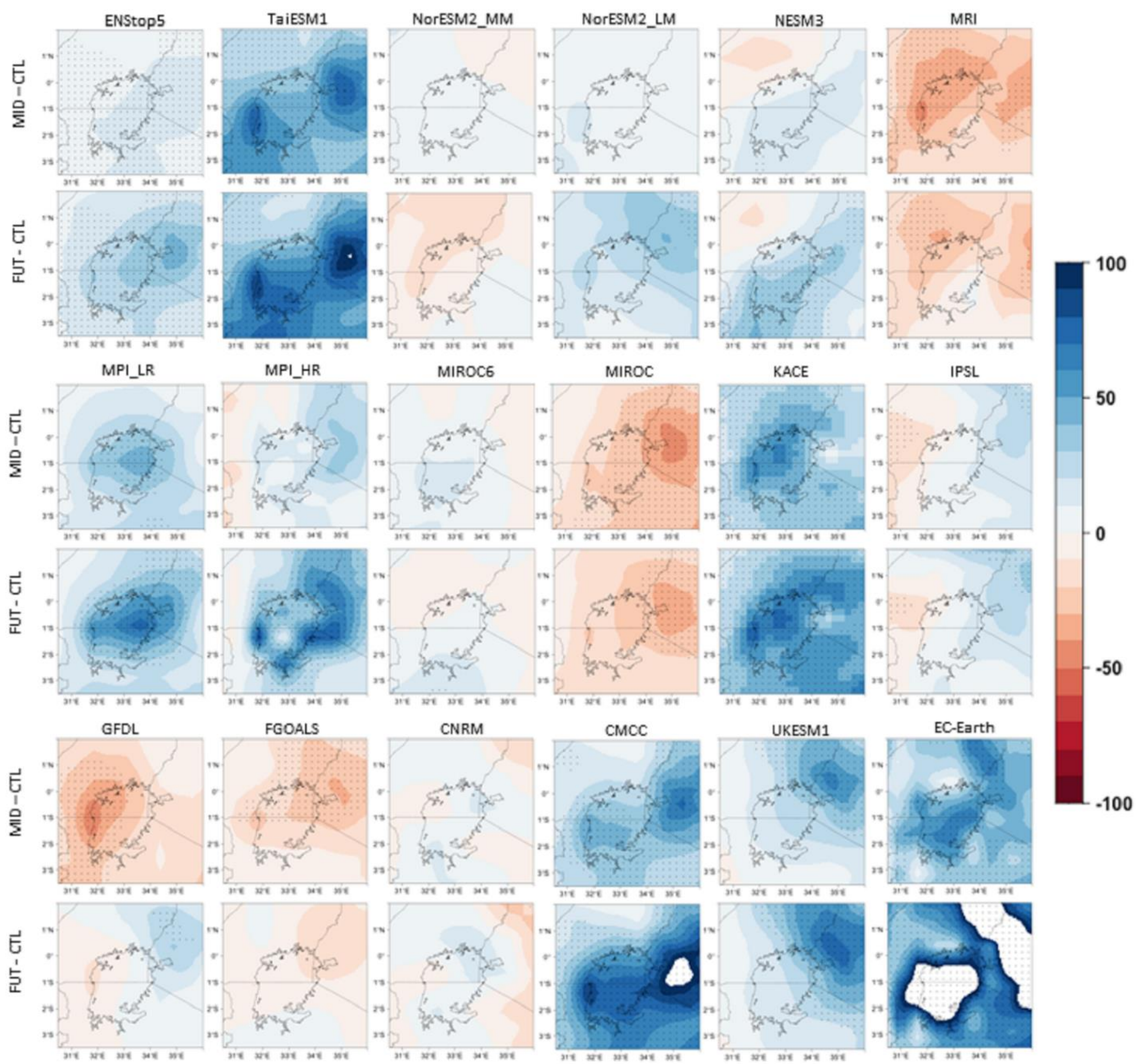

Fig. S 7: As in Fig. S 6 but for MAM

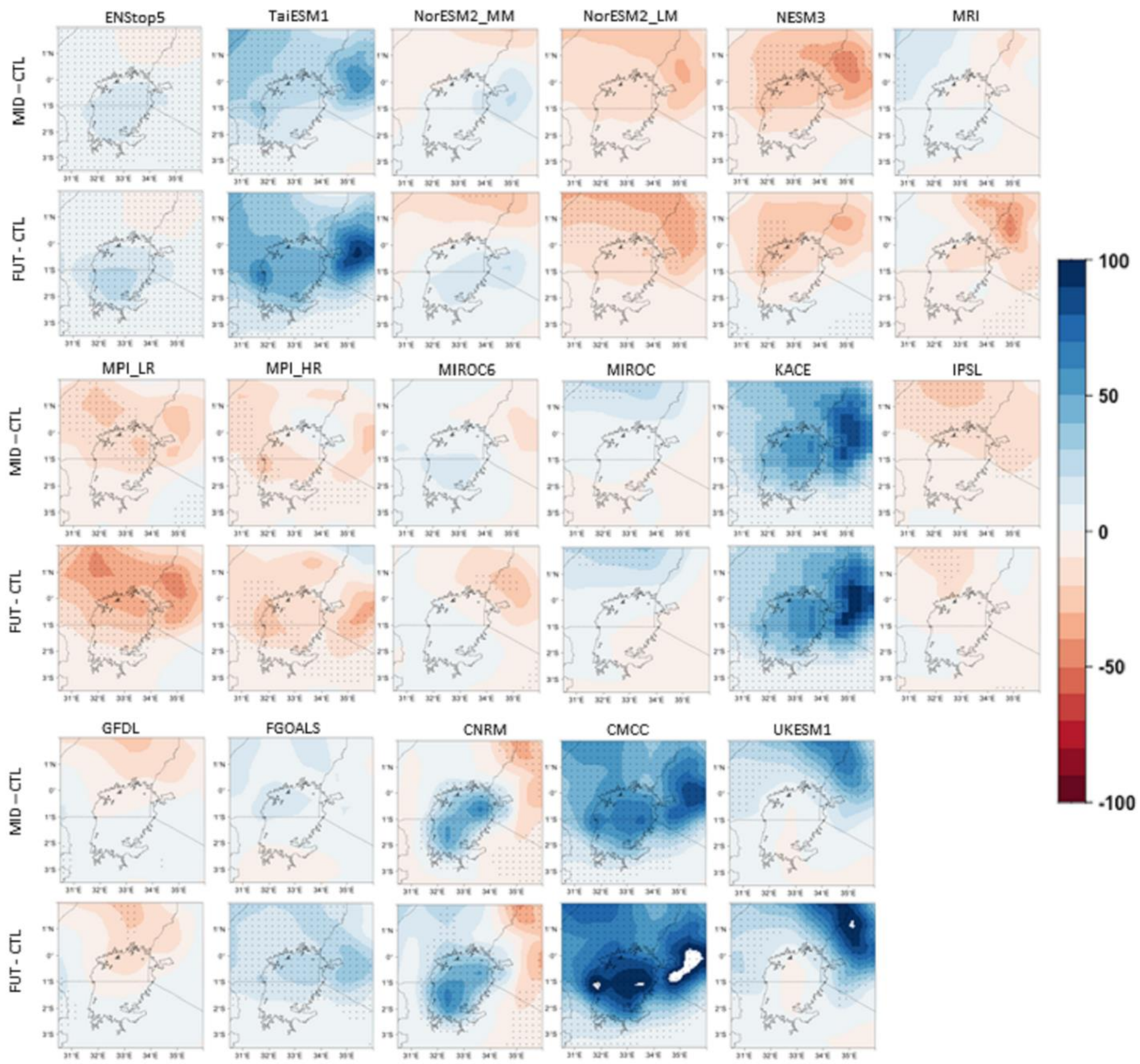

Fig. S 8: As in Fig. S 6 but for JJA

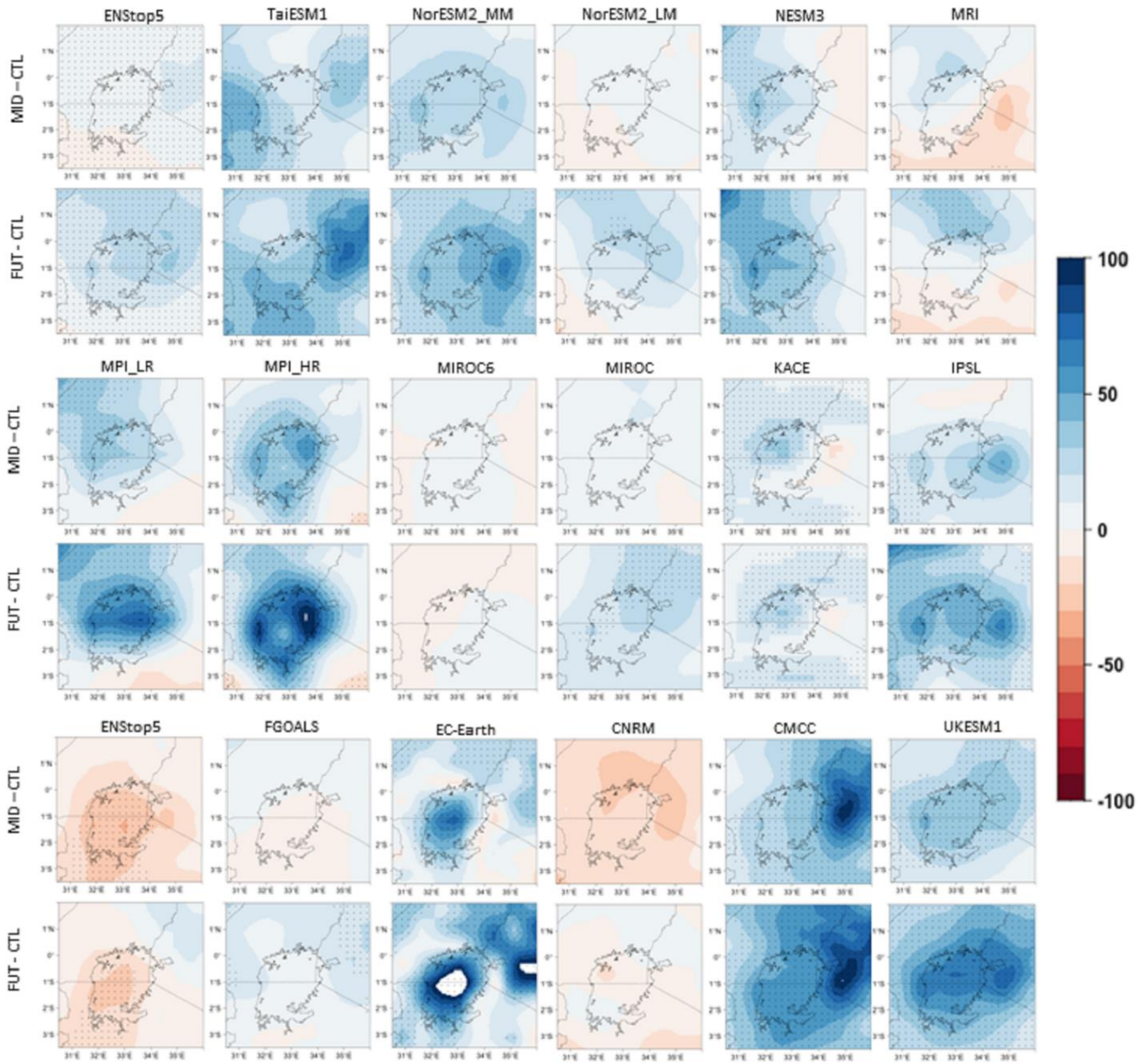

Fig. S 9: As in Fig. S 6 but for OND

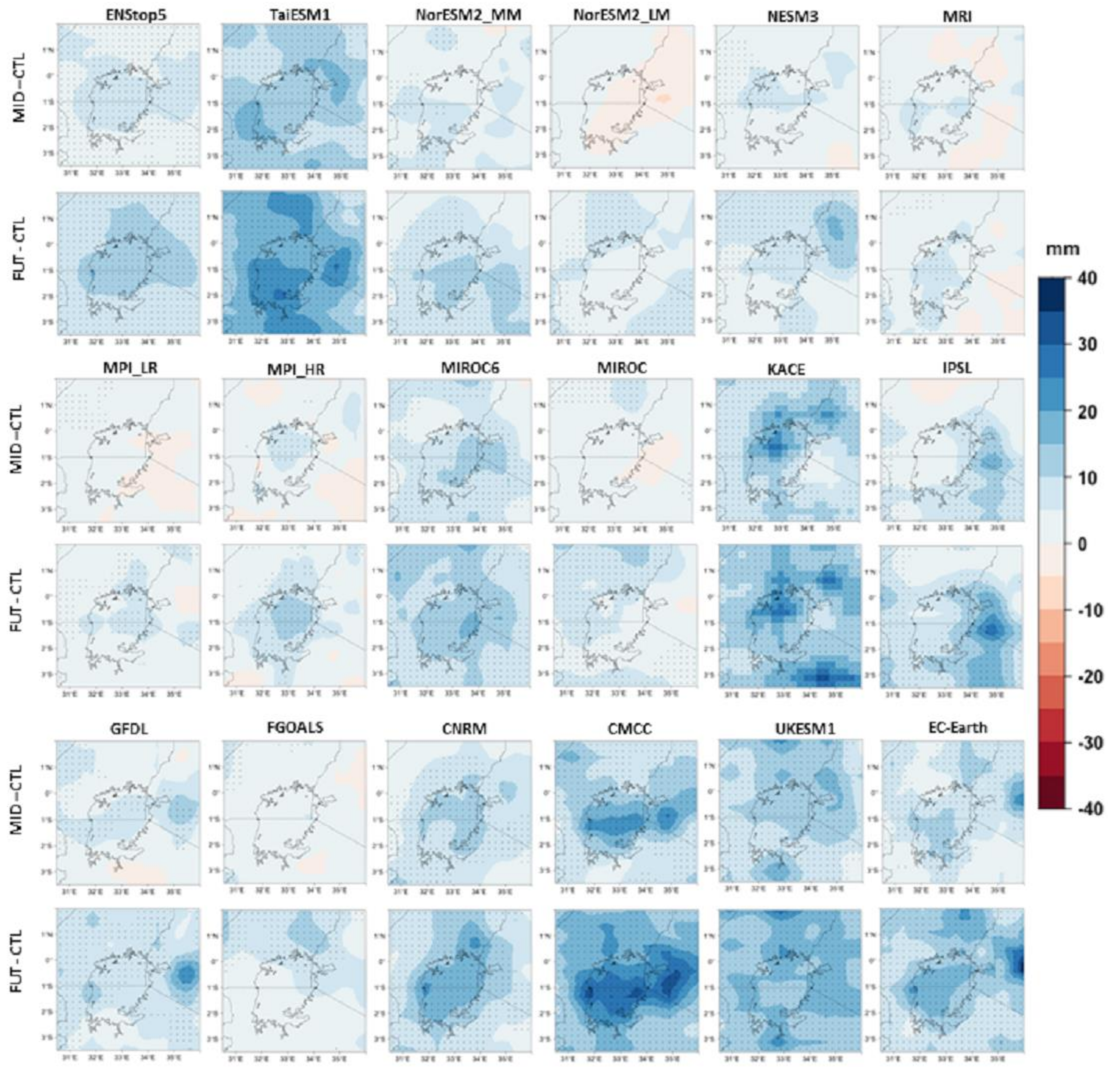

Fig. S 10: Changes in the maximum 5-day precipitation (Rx5day) climatology for the period 2040-2069 (MID-CTLl) and 2070-2099 (FUT-CTL) relative to the 1985-2014 period (CTL), for all models and an ensemble mean for the top-five models (ENStop5). Stippling shows significant values at 99% confidence level. All units are in mm

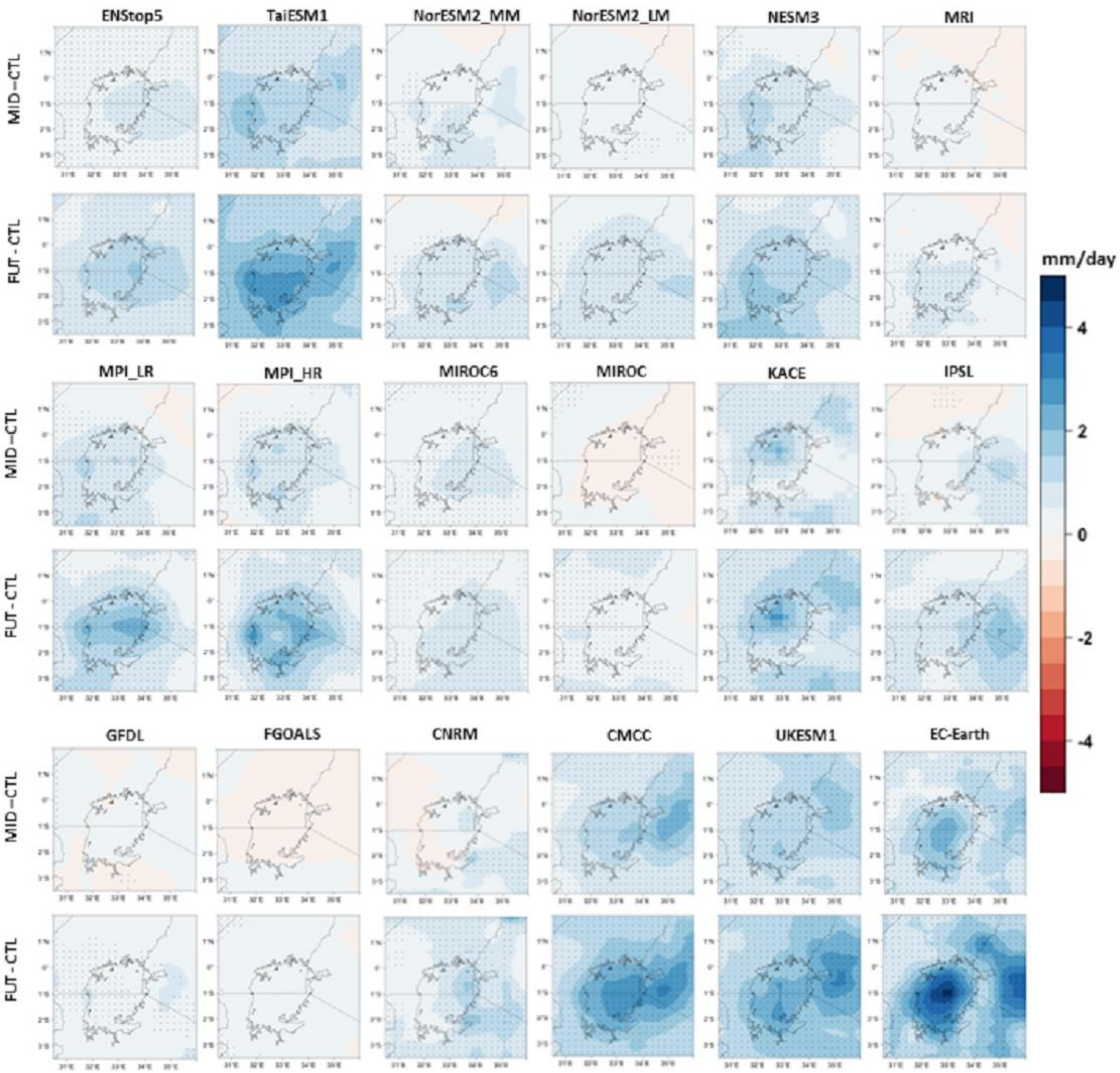

Fig. S 11: As in Fig. S 10 but for SDII

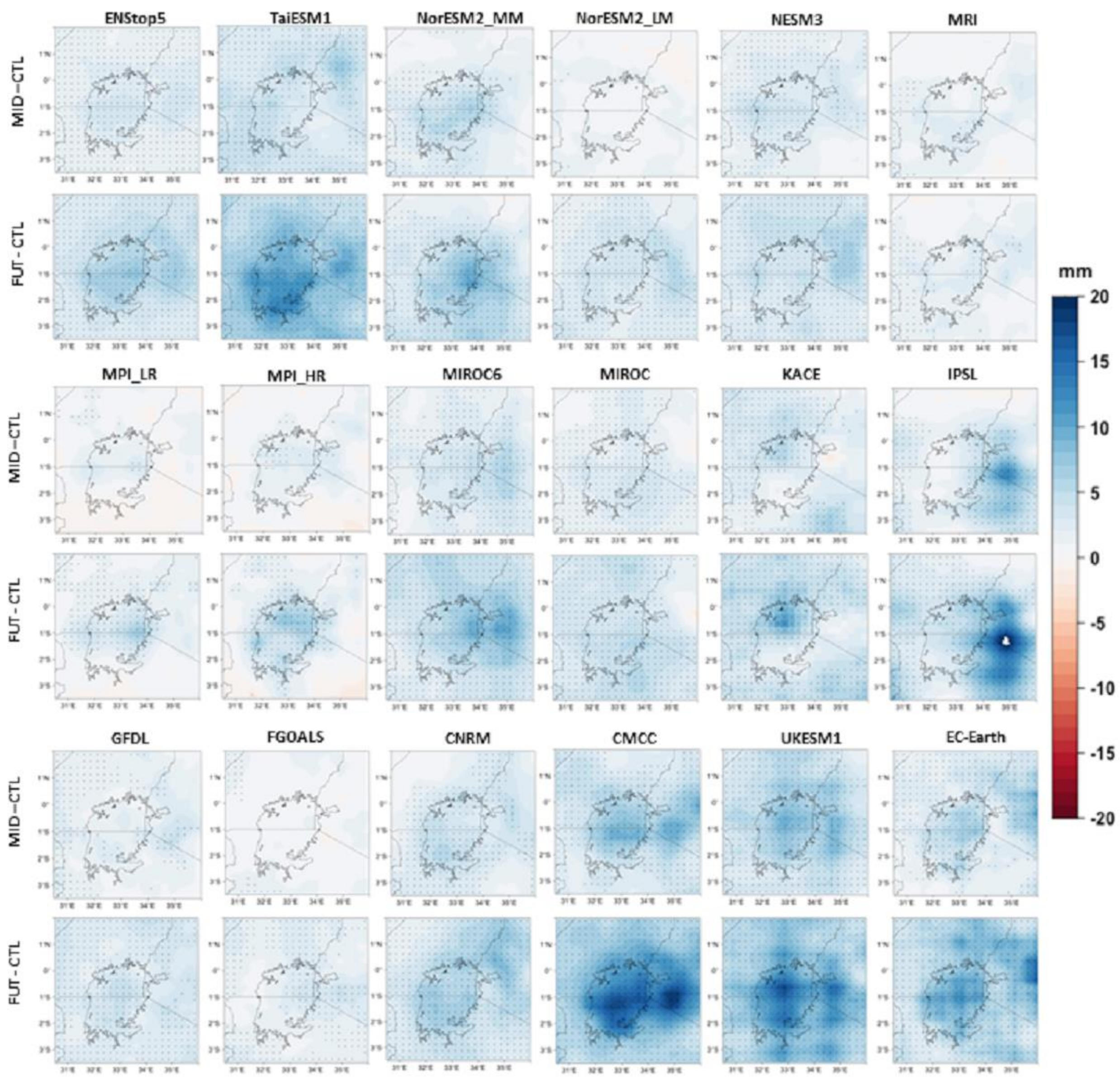

Fig. S 12: As in Fig. S 10 but for 99p90p. Units are in mm
